# Supplementary material for: Myokines May Be the Answer to the Beneficial Immunomodulation of Tailored Exercise—A Narrative Review
Source: Biomolecules. 2024 Sep 25;14(10):1205. doi: 10.3390/biom14101205 (PMC11506288; doi:10.3390/biom14101205)
Supplement: Supplementary file 1 [file biomolecules-14-01205-s001.zip › Supplementary chart.pdf]

Supplementary Table 1. The standard of exercise intensity

	Relative Intensity			Absolute Intensity	Absolute Intensity (MET) by Age			Resistance Exercise Relative Intensity
Intensity	%HRR or % VO_2R	% HR_{max}	% VO_{2max}	METs	Young (20-39yr)	Middle-aged (40-64yr)	Older (≥65yr)	% 1RM
Very light	< 30	< 50	< 37	< 2	< 2.4	< 2.0	< 1.6	< 30
Light	30-39	57-63	37-45	2.0-2.9	2.4-4.7	2.0-3.9	1.6-3.1	30-49
Moderate	40-59	64-76	46-63	3.0-5.9	4.8-7.1	4.0-5.9	3.2-4.7	50-69
Vigorous	60-89	77-95	64-90	6.0-8.7	7.2-10.1	6.0-8.4	4.8-6.7	70-84
Near-maximal to maximal	≥90	≥96	≥91	≥8.8	≥10.2	≥8.5	≥6.8	≥85

Table referred to the criteria proposed by the American College of Sports Medicine (ACSM)[186].

HR_{max}:maximal HR; %HR_{max}: percent of maximal HR; HRR: HR reserve; VO_{2max}: maximal oxygen uptake; % VO_{2max}: percent of maximal oxygen uptake; VO_2R: oxygen uptake reserve; METs: Metabolic Equivalent of Task; 1RM: One-repetition maximum.
